# From instable directional switching to controlled unidirectional operation in a nonlinear fiber ring laser

**DOI:** 10.1038/s41598-024-56506-3

**Published:** 2024-03-12

**Authors:** Alexander Hartung, Muhammad Assad Arshad, Matthias Jäger

**Affiliations:** Leibniz Institute of Photonic Technology, Albert-Einstein-Straße 9, 07747 Jena, Germany

**Keywords:** Fibre lasers, Nonlinear optics

## Abstract

We investigate a new phenomenon, where a reciprocal fiber ring laser switches from bidirectional to unidirectional operation above a certain pump power threshold. Significant simplifications regarding earlier experiments are presented, which for the first time allow the identification of individual nonlinear effects. We highlight the unique role of stimulated Raman scattering in triggering unidirectional operation, and that additional conditions apply. The threshold is reduced from 30 to 3.8 W, which eases potential applications.

## Introduction

In our last publication we demonstrated a unique phenomenon, where a simple reciprocal fiber ring laser without any directional constraints switched reliably from bidirectional to unidirectional operation above a certain pump power threshold^1^. From an application perspective it appears very attractive to get a ring laser operating in a single direction without the need of a dedicated, optically non-reciprocal element or layout like e.g. an isolator^2,3^, a single-sided stepped fiber taper^4^, a theta-cavity design^5–8^, or via mode-locking by nonlinear polarization evolution^9^, because this typically imposes additional constrains like limited power handling or wavelength flexibility. Before this new phenomenon can be optimized for specific applications, the underlying physical relationships must be revealed. This paper provides an important step in this direction.

We shortly summarize the most relevant facts from our last publication^1^, which is to the best of our knowledge the first publication covering this topic. The setup in question was a simple cladding-pumped fiber ring laser consisting of a pump signal combiner, 6 m Yb-doped active fiber (Nufern SM-YDF-5/130), 1 km single-mode fiber (Corning HI1060) and a 0.01% tap coupler for monitoring the circulating powers and spectra. When reaching the laser threshold, the laser started to emit bidirectionally in both the clockwise and the counterclockwise direction with similar power. This is the expected behavior and it is also generally expected to stay this way independent of the pump power. Surprisingly, we observed another threshold-like behavior at 30 W pump power, where the laser switched to unidirectional operation. At this time, we observed that there is a probability attached, and that each direction can occur as the remaining one. For this reason, we termed this phenomenon ‘direction instability’.

We also investigated the dependence of this other, second threshold on the length of the passive fiber and found, that it follows a power-times-length scaling. This indicated a nonlinear origin and led us to the hypothesis that the reason for unidirectional operation is a nonlinear effect taking place in the passive fiber, discriminating one direction over the other by disproportional removal of power off the laser wavelength, eventually leading to its termination. While the very broad, feature-rich and continuum-like spectrum spanning from 1100 nm to above 1600 nm was an advantage for the initially targeted broadband fiber light source, this made it impossible to distinguish individual effects and hence we were not able to further specify the nonlinear effect in question.

In the current manuscript we introduce a dedicated spectral filter section to simplify the spectrum down to individual spectral features. This allows a much clearer picture about what is or is not happening in the passive fiber in terms of nonlinear effects. We show that it is simulated Raman scattering (SRS) which finally triggers the second threshold corresponding to unidirectional operation, but we also show that there is no directional discrimination apparent, which contrasts our initial hypothesis based upon a disproportional nonlinear power removal off the laser wavelength.

## Experimental setup

**Figure 1:**
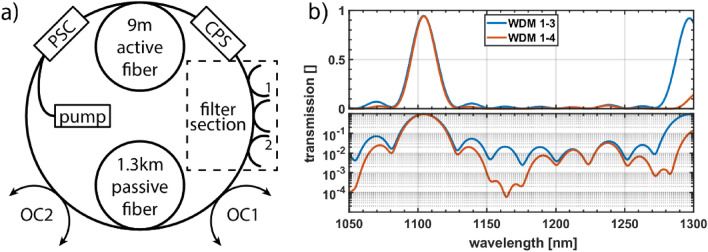
(**a**) The ring laser setup including a 976 nm pump diode, a power signal combiner (PSC), 9 m Yb-doped fiber, a cladding power stripper (CPS), a filter section made of a series of WDMs, 1.3 km passive fiber, and two output couplers (OC1 and OC2) for characterization. Two ends of the filter section are labeled (1 and 2) for later reference in Sect. “Seeding Raman”. (**b**) Transmission of the filter section in two different configurations in linear scale (top) and logarithmic scale (bottom). Note the additional loss around 1163 nm for the configuration with WDMs 1 to 4 compared to the one with WDMs 1 to 3 for additional suppression of SRS.

The fiber ring laser is rather similar to the initial one presented in^1^. It is comprised of the pump-signal-combiner, the active fiber Nufern SM-YDF-5/130, the passive fiber Corning HI1060 and now two output couplers enclosing the passive fiber for a closer look into its internal events (Fig. 1a). The length of the active fiber was increased from 6 m to 9 m to reduce the residual pump power and make life easier for the cladding power stripper. The output couplers now have an increased output coupling ratio of 1% (up from 0.01%) to make calculations from values measured outside the ring to values occurring inside the ring more robust.

Most importantly, we added a filter section. This is a series of fused fiber wavelength division multiplexers, all with a high transmission at the laser wavelength (1100 nm) and a tailored output coupling ratio elsewhere. This allowed us to effectively prevent the generation and propagation of selected spectral regions and to investigate their relation to the occurrence of unidirectional operation. In total we used 4 different WDMs with design wavelengths of 1100/1200 nm, 1100/1150 nm, 1100/1125 nm, and with 1106/1163 nm. The first three are intended to be used together and they cause a broad and flat outcoupling starting at 1120nm and above (Fig. 1 right). The fourth WDM is specifically designed with a period of 57 nm equal to the 13 THz SRS shift for a targeted suppression of SRS.

## Operation at restricted spectrum

**Figure 2:**
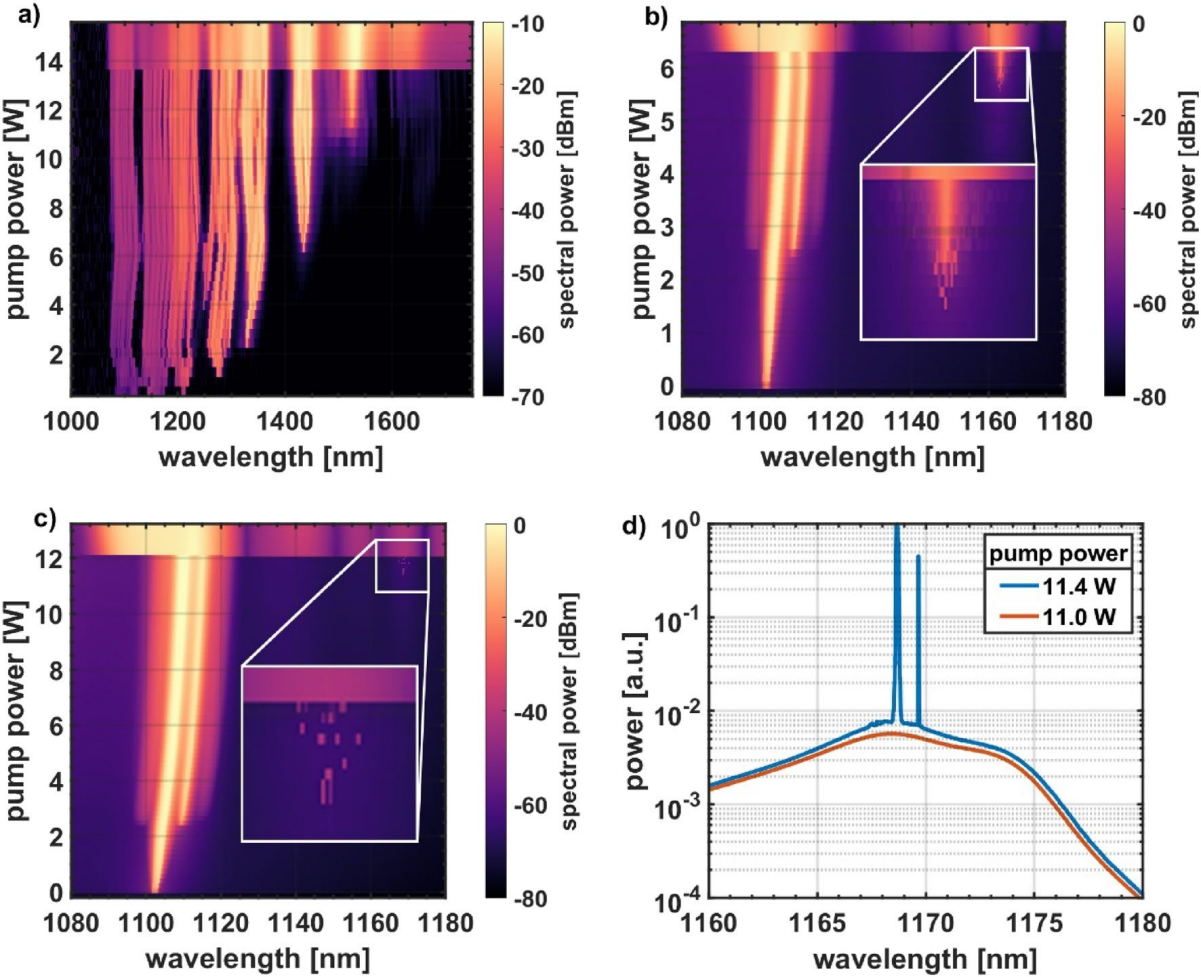
Spectral evolution for the preferred counter clockwise direction at the entrance of the passive fiber with (**a**) no filter stage included in the cavity (broad spectrum with 6 Stokes orders prior to NUO), (**b**) filter stage constituted of WDMs 1–3 (a single Stokes peak prior to NUO and prevention of broadening of the laser wavelength), (**c**) WDMs 1–4 (SRS and NUO retardation to 12 W pump power). NUO is present in the upper parts of the figures accompanied by a much broader spectrum compared to bidirectional operation for lower pump powers. Note the different y-axes. The inset in (**b,c**) highlight SRS right below the NUO threshold. In (**d**) the Raman spectrum is shown at the threshold to lasing.

The inclusion of WDMs in the ring had the desired effect on the spectrum as seen in Fig. 2. The initial supercontinuum-like spectrum (Fig. 2a) was reduced to three spectral features by WDMs 1 to 3, two around the laser wavelength at 1106 nm and 1112 nm and one at the first SRS peak at 1163 nm (Fig. 2b). This is a huge help to exclude potential nonlinear effects to trigger unidirectional lasing. No power is required in the anomalous dispersion regime (> 1440 nm for Corning HI1060) for e.g., soliton-related effects. No power is required in the vicinity of the zero-dispersion wavelength for phase-sensitive effects like four-wave-mixing. Higher order Stokes generation and a strong broadening of the laser wavelength are not relevant to trigger unidirectional operation, as well.

We also observed now a consistent switch to always the same direction (counterclockwise) in contrast to earlier experiments with a probability for both directions to occur as the final one. For this reason, we now term this phenomenon ‘nonlinear unidirectional operation’ (NUO) in the context of this publication and not ‘direction instability’. But both terms refer to the same phenomenon.

There was still some SRS present prior to the switch to unidirectional operation (inset of Fig. 2b), so we tested, whether we can push SRS past the NUO threshold. For this reason, SRS was dedicatedly suppressed by the addition of WDM 4 into the cavity and the resulting spectral evolution is shown in Fig. 2c). The threshold now strongly increased from 6.2 W to 12.0 W.

On closer inspection, you still see the first strong SRS signals right before the NUO threshold (inset of Fig. 2c). This is the spiking spectrum of a Raman laser at threshold (Fig. 2d). The Raman gain $\frac{{P_{out} }}{{P_{in} }}$ is connected to the pump power $P_{0}$ through equation $\frac{{P_{out} }}{{P_{in} }} = \exp \frac{{g_{r} P_{0} L}}{A}$. Here, $g_{r} = 1\cdot10^{ - 13} \;\frac{{\text{m}}}{{\text{W}}}$ is the Raman gain coefficient, $L = 1.15\;{\text{km}}$ is the effective length of the passive fiber, and $A = 33.2\;\upmu {\text{m}}^{2}$ is its data sheet mode area. This equation is used to estimate the required intra-cavity powers at the entrance of the passive fiber to cancel the suppression of the filter section. A gain of 36 dB to cancel the suppression of WDMs 1 to 4 requires approx. 2.4 W intra-cavity power and a gain of 16 dB to cancel the suppression of WDMs 1 to 3 requires approx. 1.1 W intra-cavity power. These power values are slightly lower than the measured ones (3.1 W and 1.8 W, respectively) and reasonably suggest the occurrence of a Raman laser.

This observation suggests, that the presence of a strong SRS signal, or maybe more specifically the conditions of a Raman laser, where there is enough Raman gain to cancel roundtrip losses, is a necessary condition for NUO to occur. Note, that in Fig. 2a,b the Raman laser is established considerably before the NUO threshold, indicating that the Raman laser is only a necessary condition and not a sufficient one.

## Seeding Raman

If NUO is triggered by SRS we should be able to influence the effect with an external seed at the SRS wavelength around 1163 nm. We added a laser diode at port 1 of the filter section (as shown in Fig. 1) and monitored the output power progression for various seed powers. Keep in mind, that the 1163 nm signal (Raman seed plus all occurring gain) is mostly coupled out of the ring after one round-trip when it reaches the filter stage, and that the original Raman seed power is restored at the last port of the filter stage. Also keep in mind that only one direction, the already preferential counterclockwise one is seeded, and that virtually no Raman signal is propagating in the clockwise direction.

**Figure 3:**
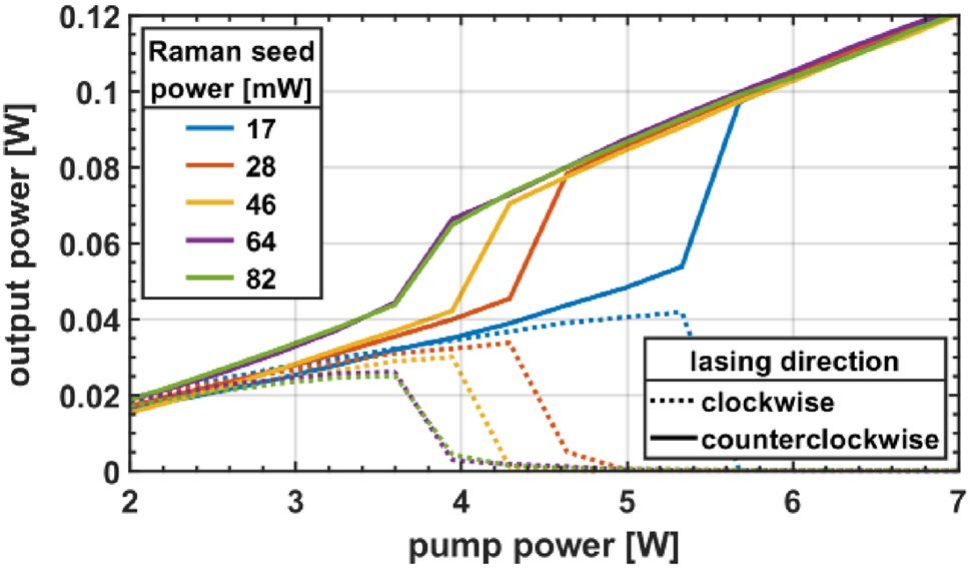
Output power progression for various Raman seed powers. The NUO threshold can be reduced with increasing seed power down to a certain limit.

The result is shown in Fig. 3. Without the 1163 nm seed the NUO threshold was around 12 W pump power. With the 1163 nm seed the threshold reduced to 3.8 W. This clearly proves, that SRS is responsible for triggering NUO. The NUO threshold could not be reduced below 3.8 W with higher SRS seed powers. This again highlights, that the SRS signal is only a necessary condition but not a sufficient one to trigger NUO und that there are additional conditions to uncover.

In another experiment, we omitted the external seed diode but changed an angle-terminated output of the filter section to a straight, perpendicular cleave. This changes the function of the filter section. Instead of completely suppressing the Raman wavelength for both directions, the filter section now effectively provides a Raman seed for one direction, established by a Fresnel reflection of the Raman signal of the counter direction.

Providing a straight cleave at port 1 (confirm Fig. 1) resulted in seeding the counterclockwise direction and had a similar effect as placing the seed diode at this position. This simple change alone reduced the NUO threshold from 12.0 W down to 7.8 W. In another experiment we provided a straight cleave at port 2 only, this time seeding the opposite, clockwise direction. As a result, the laser now consistently switched into this clockwise direction. The threshold has not changed in this case but stayed at a similar level of 12 W, as with angle-terminated fiber ends. Both experiments highlight the importance of SRS to trigger NUO and also provide a surprisingly simple practical mean to control the final direction of the ring laser.

**Figure 4:**
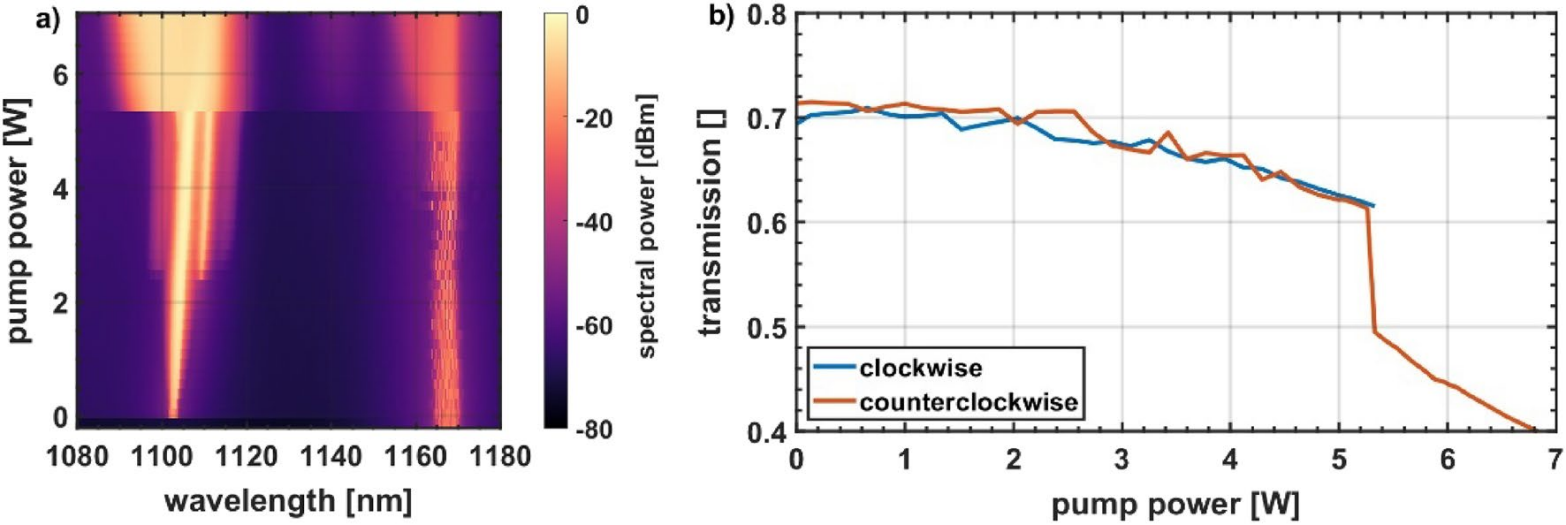
NUO experiments with a constant Raman seed of 17 mW. (**a**) Counterclockwise spectral evolution at the entrance of the passive fiber and (**b**) transmission of the laser wavelength (excluding power > 1130 nm) through the 1.3 km passive fiber. Both directions experience the same loss and hence contribute equally to the gain of the single direction Raman signal.

Next, we wanted to clarify, whether any directional imbalance in the sense of a disproportional power transfer off the laser wavelengths around 1100 nm is created in the passive fiber. Therefore, input and output spectra and powers of the passive fiber for both directions were measured. Based on these measurements the transmission of the laser wavelength (spectral components < 1130 nm) along the passive fiber was calculated. This experiment was conducted with a constant SRS seed power of 17 mW. The results are presented in Fig. 4.

For both directions, the transmission starts around 70% at low pump powers. This is reasonably close to the linear loss of the 1.3 km passive fiber with 1 dB/km used here, as we do not expect any additional nonlinear loss at low pump power. Transmission slowly drops for higher pump power since increasingly more power can be effectively transferred via SRS to 1163 nm. But most importantly, we do not see any sign of directional discrimination up to the second laser threshold, which is around 5.3 W in this case. Both, the clockwise and the counterclockwise laser signal equally reduce their power and contribute to the single direction SRS signal. The drop at and after the second threshold is caused by additional power conversion through SRS.

We did a similar experiment where we kept the pump power constant at 4.3 W and varied the Raman seed power from 10 to 100 mW and observed the same behaviour. While the absolute transmission of the laser wavelength changed depending on the seed power, both directions always suffered the same loss.

## Discussion and conclusion

This paper focuses on possible nonlinear effects occurring in the passive fiber section of the ring laser eventually leading to unidirectional operation. We revealed that the formation of a Raman laser precedes the switch to unidirectional operation. This has interesting implications. The Raman gain is isotropic, signifying that co- and counter-pumped SRS share the same Raman gain value. As a result, this nonlinear effect depletes both lasing directions equally and is not capable of discriminating a specific one. This is also in line with our observation, that both propagation directions exhibit the same transmission through the passive fiber.

We also demonstrated, that SRS is only a necessary condition and that additional conditions apply, and we now expect these to be located in the active fiber. Currently, the most obvious directional dependence is the level of SRS. Assuming a similar level of SRS being generated for both directions along the passive fiber, this SRS signal experiences a different amount of linear loss depending on the propagation direction before it reaches the active fiber, because the cavity is not symmetric component-wise.

In our latest experimental configuration, the full SRS signal reaches the active fiber for the clockwise direction, which is finally shut off, while the SRS signal is reduced by 4 orders of magnitude for the counterclockwise direction, which finally survives. Essentially, the active fiber experiences two seed signals (1100 nm and its SRS signal at 1160 nm) for one direction and only one seed signal for the other direction. We also expect this directional dependence to be the reason for the consistent switch to always the same direction for 100 + experiments, in contrast to earlier experiments without filter stage, a similar feedback per direction from the passive fiber to the active fiber and a probability for each direction to occur as the final one.

Future work will focus on experiments with an external seed targeting the active fiber. If the passive fiber only serves the purpose of providing a second seed signal, maybe it can even be omitted in such a case. Note, that the SRS signal of 1160 nm lies within the gain bandwidth of Yb and we expect this to be a necessary condition of the second seed signal, to interact with the active fiber and to have an impact on its emission. Therefore, other seed wavelengths might be suitable, as well. This has to be clarified in future investigations.

Besides these scientific investigations, the phenomenon has evolved from a directional instability to a deterministic switch to a predefined direction. In addition, the threshold has been lowered considerably from initially 30 W down to 7.8 W for cavities with self-seeded SRS by perpendicular instead of angled-terminated fiber ends, and values as low as 3.8 W with a proper external Raman seed. These properties increase the attractiveness for potential future applications.

## Data Availability

Data underlying the results presented in this paper are not publicly available at this time but may be obtained from the authors upon reasonable request.
